# Exosomes in Dermatology: Emerging Roles in Skin Health and Disease

**DOI:** 10.3390/pharmaceutics17050600

**Published:** 2025-05-02

**Authors:** Salman Bin Dayel, Ramadan S. Hussein

**Affiliations:** Department of Dermatology, College of Medicine, Prince Sattam Bin Abdulaziz University, Al-Kharj 16273, Saudi Arabia; dr_bindayel@hotmail.com

**Keywords:** exosomes, skin health, skin homeostasis, dermatological conditions

## Abstract

**Background/Objectives:** Exosomes, nanosized vesicles secreted by diverse cell types, have emerged as critical mediators of intercellular communication, tissue repair, and disease pathogenesis. Their roles in dermatology are increasingly recognized, influencing skin health and the progression of various dermatological conditions. This review aims to explore the biogenesis, composition, and mechanisms of exosome uptake in skin cells and their implications in dermatological research and clinical practice. **Methods:** A comprehensive review of the existing literature was conducted to elucidate the biological composition of exosomes, their roles in skin homeostasis, and their involvement in processes, such as wound healing, tissue regeneration, and barrier function maintenance. This review also examined the diagnostic and therapeutic potential of exosomes in conditions such as psoriasis, eczema, acne, and skin cancer. **Results:** Exosomes were found to contain intricate compositions, including proteins, lipids, nucleic acids, and bioactive molecules, crucial for maintaining skin homeostasis. They demonstrated significant roles in modulating wound healing and skin regeneration. Emerging evidence highlights their involvement in dermatological conditions and their potential as diagnostic biomarkers and therapeutic agents. Exosome-based approaches hold promise for advancing disease management, although challenges remain in translating these findings into clinical applications. **Conclusions:** Exosomes represent a promising frontier in dermatology, with the potential to revolutionize the understanding, diagnosis, and treatment of skin-related disorders. Despite the challenges, their complexity and versatility underscore their potential in developing personalized skin health strategies. Further research is warranted to address the existing gaps and harness the full therapeutic potential of exosomes in dermatological applications.

## 1. Introduction

The skin, our body’s largest organ, serves essential functions in protection, thermoregulation, and sensory perception, while also influencing our self-esteem and emotional well-being. Given its multifaceted significance, maintaining skin health and managing dermatological conditions are paramount [1]. Recent years have witnessed a transformative shift in our comprehension of intercellular communication, attributed to the discovery of exosomes—small, membrane-bound vesicles secreted by various cell types [2].

Initially considered cellular waste disposers, exosomes have gained prominence as potent mediators of cell-to-cell communication, impacting health and disease across organ systems. Within dermatology, exosomes have emerged as versatile actors crucial for modulating cellular responses, regulating tissue repair, and participating in immune responses [3]. This review aims to unravel their complex involvement in dermatology, exploring their biogenesis, cargo loading mechanisms, and dynamic interplay with skin cells.

The comprehensive examination encompasses the roles of exosomes in tissue repair, wound healing, and maintaining the skin’s barrier function [4]. Beyond physiological functions, exosomes play roles in dermatological diseases, from inflammatory disorders like psoriasis to challenges in managing conditions such as acne and skin cancer [5]. In diagnostics and therapy, exosomes offer a promising frontier, providing a non-invasive glimpse into internal processes and potential targeted therapeutic delivery [6].

Ongoing research and clinical trials indicate that exosome-based interventions may revolutionize approaches to skin health and dermatological condition management [7]. This review not only uncovers exosomes’ roles in physiological and pathological skin processes but also contemplates the challenges and opportunities in harnessing their potential. Future horizons of exosome research are discussed, identifying areas ripe for further investigation.

The complex world of exosomes holds promise for advancing our understanding, diagnosis, and treatment of skin-related conditions. By exploring their role in dermatology, this review aims to pave the way for innovative and personalized strategies to safeguard and enhance skin health in the evolving landscape of medical science.

## 2. Exosome Biogenesis and Function in Skin and Other Tissues

Exosomes, small extracellular vesicles with a diameter typically ranging from 30 to 150 nanometers, are a subclass of extracellular vesicles that play crucial roles in intercellular communication and the transport of bioactive molecules. They are secreted by a variety of cell types and have gained significant attention for their diverse roles in health and disease, including their pivotal functions in dermatology [8].

Exosome biogenesis is a complex and precisely regulated process that occurs within the endosomal system of cells and involves endocytosis, maturation into multivesicular bodies (MVBs), cargo incorporation, MVB fates, and exosome secretion. The endocytosis process begins with the invagination of the plasma membrane to form early endosomes. These early endosomes contain extracellular materials that are internalized from the cell’s surroundings. Early endosomes mature into late endosomes or multivesicular bodies (MVBs) through a series of membrane transformations [3]. This maturation process involves the inward budding of the endosomal membrane, resulting in the formation of intraluminal vesicles (ILVs) within MVBs. These ILVs are the precursors of exosomes. During the maturation of MVBs, specific proteins, lipids, nucleic acids, and other bioactive molecules are selectively incorporated into ILVs. These molecules constitute the cargo of exosomes and are typically protected from degradation. MVBs can either fuse with lysosomes for cargo degradation or they can traffic to the cell membrane for exosome secretion. MVBs that are destined to become exosomes move toward the cell membrane and fuse with it, releasing the ILVs into the extracellular space. Once released, these ILVs are referred to as exosomes [5,8].

Exosomes exhibit distinctive characteristics, including their small size, making them smaller than other extracellular vesicles like microvesicles and apoptotic bodies. Enclosed by a lipid bilayer membrane, exosomes’ internal contents are separated from the extracellular environment, comprising various lipids, such as cholesterol, sphingolipids, and phospholipids. They contain a unique set of proteins crucial for exosome biogenesis, cargo sorting, and cellular communication, with common markers like CD9, CD63, CD81, Alix, and TSG101 used for isolation and identification. Exosomes carry diverse cargo, including proteins, lipids, nucleic acids (DNA, RNA), and bioactive molecules, with specific compositions varying based on the cell of origin and cellular context. Notably stable, exosomes are suitable for transportation in body fluids, protecting their cargo from degradation [5,9,10].

Exosomes interact with target cells by binding to specific cell surface receptors or directly fusing with the target cell’s plasma membrane. This interaction facilitates the transfer of their cargo, consisting of signaling molecules, genetic material, and proteins, influencing recipient cells and modulating various cellular processes. As essential mediators of intercellular communication, exosomes play a pivotal role in dermatology, impacting skin biology, wound healing, immune responses, and the pathophysiology of dermatological diseases [11].

Exosomes originate from endosomes, which are derived from the plasma membrane. As early endosomes mature into late endosomes, inward budding occurs, forming multivesicular bodies (MVBs) that contain numerous intraluminal vesicles (ILVs). MVBs can either undergo degradation via lysosomes or fuse with the plasma membrane, releasing ILVs as exosomes. In contrast, microvesicles form directly through the outward budding of the plasma membrane.

## 3. Exosome Composition

Exosomes, small extracellular vesicles secreted by diverse cell types, possess a complex and diverse composition integral to their multifaceted functions in intercellular communication and dermatological processes. The constituents comprising the exosome cargo include proteins, lipids, nucleic acids, and bioactive molecules [10].

Proteins within exosomes encompass tetraspanins, like CD9, CD63, CD81, and CD82, which serve as hallmarks and crucial contributors to exosome biogenesis and are commonly used as markers for exosome identification. Proteins like Alix (ALG-2-interacting protein X) and TSG101 (Tumor susceptibility gene 101) participate in intraluminal vesicle formation (ILVs) within multivesicular bodies (MVBs), aiding exosome biogenesis. Heat shock proteins (HSPs), such as HSP70 and HSP90, protect exosome cargo and may influence immune responses. Exosomes carry cell-specific proteins related to their origin, making them unique and capable of modulating target cell responses. Various enzymes, such as metabolic enzymes, and signaling molecules, like cytokines and growth factors, are often present in exosomes, contributing to their diverse functions in cellular processes [5,9].

Lipids constitute a crucial aspect of exosome composition. Cholesterol, as a fundamental component of the lipid bilayer, plays a key role in providing stability to exosome membranes. Additionally, sphingomyelin, ceramide, and various other sphingolipids are essential structural lipids within exosome membranes. Phospholipids, including phosphatidylserine, phosphatidylethanolamine, and phosphatidylcholine, represent examples of the diverse phospholipids that collectively form the lipid bilayer of exosomes [12].

Regarding nucleic acids, exosomes containa variety of RNA species, including messenger RNA (mRNA), microRNA (miRNA), long non-coding RNA (lncRNA), and other non-coding RNAs. These diverse exosomal RNAs have the capacity to transfer to recipient cells, thereby regulating gene expression and influencing various cellular functions. Although the occurrence of DNA in exosomes is less frequent compared to RNA, certain exosomes do contain fragments of genomic DNA. The exploration of the significance of exosomal DNA is an ongoing area of research [13].

Regarding bioactive molecules, exosomes can contain cytokines, including proinflammatory or anti-inflammatory types, which influence immune responses and inflammatory processes. Additionally, various growth factors, such as epidermal growth factor (EGF) and fibroblast growth factor (FGF), are frequently identified in exosomes, potentially exerting influence on cell proliferation, tissue repair, and skin regeneration. Furthermore, exosomes can transport metabolites like amino acids, lipids, and other small molecules, participating in metabolic processes upon delivery to recipient cells [2].

Exosomes consist of diverse proteins, including major histocompatibility complex (MHC)-II, integrins, cluster of differentiation (CD) markers, tetraspanins, heat shock proteins (Hsp), and Ras-related proteins (Rab), among others. They also contain various lipids, such as sphingomyelin and cholesterol. Additionally, exosomes carry nucleic acids, including microRNA (miRNA), messenger RNA (mRNA), and non-coding RNAs.

## 4. Mechanisms of Exosome Uptake

Exosomes, with their cargo of bioactive molecules, play a pivotal role in intercellular communication and can influence various processes in dermatology. However, their functions are intricately linked to how they are taken up by recipient cells [14]. Mechanisms such as clathrin-mediated endocytosis through clathrin-coated pits on the cell membrane can facilitate the uptake of exosomes. These pits bind to specific receptors on the exosome surface, leading to their internalization [15]. Caveolae are small invaginations of the cell membrane, and they can participate in exosome uptake. Exosomes may interact with caveolin molecules and enter cells through caveolae [16].

Immune cells like macrophages can phagocytose exosomes as part of their normal functions. This mechanism is particularly relevant in immune responses and inflammation in dermatology [17].

Macropinocytosis is a non-specific form of endocytosis that allows cells to internalize exosomes through the engulfment of large portions of extracellular fluid, including exosomes. Exosomes can also directly fuse with the cell membrane, releasing their cargo into the cytoplasm. This process is a rapid means of cargo delivery [18].

Some exosomes carry ligands on their surface that can bind to specific receptors on target cells. This interaction triggers internalization through receptor-mediated endocytosis. Exosomes may express glycosylphosphatidylinositol (GPI)-anchored proteins on their surface, which can interact with lipid rafts on the recipient cell membrane, leading to exosome uptake [15,19].

Tunneling nanotubes (TNTs) are thin, actin-rich membranous structures that connect cells. Exosomes can traverse TNTs to reach distant cells, allowing for cell–cell communication in dermatological processes. Some cells can form micropinocytic vesicles to internalize exosomes. This process is distinct from macropinocytosis due to differences in vesicle size [20].

The specific mechanism involved often depends on the cell type, exosome cargo, and the context of the dermatological process. Additionally, exploring the regulatory factors and signaling pathways that govern exosome uptake is an area of active research, as it can provide insights into potential therapeutic strategies harnessing exosomes for skin health and dermatological treatments [19,20].

## 5. Skin Homeostasis

Homeostasis is the essential process that maintains the stability and equilibrium of the skin’s various components, ensuring its optimal function as a protective barrier [21]. The key aspects of skin homeostasis are described below.

One key aspect is epidermal barrier function, in which the outermost layer of the epidermis, the stratum corneum, plays a critical role in maintaining skin homeostasis. Composed of corneocytes embedded in a lipid matrix, it acts as a formidable barrier, preventing dehydration and protecting against external insults. Maintaining an optimal level of Trans-Epidermal Water Loss (TEWL) is essential for skin hydration. An imbalance can lead to dryness or excessive moisture loss, impacting skin health [21].

The balanced proliferation and differentiation of keratinocytes are crucial for the continual renewal of the epidermis. Dysregulation can lead to disorders such as psoriasis or skin cancers [22]. Sebaceous glands secrete sebum, which plays a role in skin lubrication and protection. Imbalances can result in conditions like acne or excessively oily skin. Sweat glands help regulate body temperature by producing sweat. Malfunctions can lead to heat-related issues or excessive sweating. Melanocytes are responsible for melanin production, which determines skin pigmentation. Skin homeostasis in this context involves maintaining even pigmentation and preventing conditions like hyperpigmentation or vitiligo [23].

Skin hosts various immune cells that contribute to surveillance and protection against pathogens. Disturbances in immune homeostasis can lead to inflammatory skin conditions [24]. Exosomes participate in intercellular communication, contributing to the regulation of skin homeostasis. They transport bioactive molecules that can influence cell behavior [2]. Maintaining a balanced skin microbiome is essential for skin health. Disturbances can lead to conditions such as dermatitis or skin infections [23].

Skin homeostasis also encompasses the ability of the skin to repair itself after injury. Efficient wound healing processes are crucial for restoring skin integrity. Skin homeostasis is affected by the aging process, resulting in changes like decreased collagen production and altered barrier function [25,26].

## 6. Dermatological Conditions

Dermatological conditions encompass a wide range of disorders affecting the skin, hair, nails, and mucous membranes. These conditions arise from various factors, including genetics, infections, allergies, autoimmune responses, and environmental influences [27]. Understanding these conditions is crucial for accurate diagnosis and effective treatment. The Table 1 below provides a detailed overview of common dermatological conditions, their characteristics, and underlying causes.

Understanding these dermatological conditions, their causes, and clinical presentations is essential for healthcare providers to make accurate diagnoses and develop appropriate treatment plans. Additionally, ongoing research in dermatology aims to advance our knowledge of these conditions and improve therapeutic approaches.

## 7. Exosome-Based Therapies

Exosomes, nanosized extracellular vesicles secreted by cells, have emerged as promising tools for innovative therapeutic approaches across various medical fields, including dermatology. The unique properties of exosomes, such as their ability to carry bioactive molecules and facilitate intercellular communication, have opened up new avenues for developing novel treatments for dermatological conditions. These are some potential exosome-based therapies in the field of dermatology.

Exosomes derived from stem cells, like mesenchymal stem cells (MSCs), contain growth factors and cytokines fostering tissue regeneration, suggesting promise in expediting wound healing, minimizing scarring, and revitalizing aging skin [36,37].

Demonstrating anti-inflammatory properties, exosomes can modulate immune responses, offering the potential to manage inflammatory skin conditions such as psoriasis, eczema, and dermatitis [6,38].

In the regulation of melanin production, exosomes may hold promise in addressing pigmentation disorders like vitiligo or hyperpigmentation [39].

Exosomes housing growth factors and signaling molecules show potential in stimulating hair follicles and promoting hair growth, potentially benefiting individuals with alopecia or other hair disorders [40].

Engineered exosomes carrying therapeutic agents offer enhanced targeted delivery, a prospective application for treating skin cancer or localized skin infections in dermatology [7].

Exosomes derived from fibroblasts and keratinocytes have an impact on extracellular matrix components vital for skin structure and elasticity, suggesting their utility in treating conditions like scars and stretch marks [36].

Exosomes have demonstrated efficacy in promoting collagen synthesis, reducing oxidative stress, and enhancing wound healing. Clinical trials suggest their promising role in anti-aging therapies, with exosome-infused skincare products showing notable improvements in skin elasticity and hydration [36,41].

Tailoring exosome-based therapies for individual patients provides customization potential, particularly advantageous in dermatology given the variability in skin conditions among individuals. Moreover, considered generally safe with minimal side effects compared to traditional therapies, exosome-based treatments present attractive options, especially in cases requiring long-term use for dermatological conditions [41].

## 8. Methods of Exosome Preparation and Commercial Applications

Exosome isolation techniques include ultracentrifugation, size-exclusion chromatography, and polymer-based precipitation [42,43]. Commercially, companies such as [Rion Aesthetics] and [Snow Fox Skincare] have developed exosome-based skincare products and therapeutic applications [44]. A comprehensive list of key exosome-based products and their clinical applications is provided in Table 2 [45].

## 9. Genetic Transfection Using Exosomes vs. Traditional Methods

Exosome-mediated gene delivery presents several advantages over conventional viral and non-viral transfection techniques, including higher biocompatibility, lower immunogenicity, and enhanced targeting efficiency. Table 3 compares these advantages with traditional transfection methods.

Exosomes offer higher biocompatibility, lower immunogenicity, and improved targeting efficiency compared to traditional methods. Viral transfection provides high gene delivery efficiency but poses risks of immune responses and genomic integration. Non-viral transfection methods are widely used but may suffer from lower stability and targeting efficiency. Exosome-mediated gene delivery is a promising safer alternative with potential applications in dermatology and regenerative medicine [53,55].

## 10. Patent Landscape of Exosome-Based Products

Recent advances in exosome technology have led to numerous patents globally. These patents cover innovations in exosome isolation, modifications for targeted drug delivery, and applications in dermatology. A summary of major patents in the field is presented in Table 4 [56,57,58,59,60,61,62].

Patents span various applications, including anti-aging, wound healing, and inflammatory skin conditions. Innovations focus on enhancing exosome isolation techniques and optimizing their therapeutic potential. Targeted drug delivery using engineered exosomes is an emerging frontier in dermatological research [62].

## 11. Future Directions

The utilization of exosome-based therapies in dermatology has shown remarkable promise, but the journey toward their widespread clinical application continues to unfold. To pave the way for a future where exosomes play an integral role in dermatological treatments, several key directions warrant exploration and development, as shown in Table 5 [41,63,64].

## 12. Conclusions

Exosomes, small extracellular vesicles secreted by various cell types, have emerged as powerful mediators of intercellular communication, holding significant potential in the field of dermatology. Their pivotal roles in skin homeostasis, wound healing, immunomodulation, and tissue regeneration make them intriguing candidates for innovative dermatological therapies. Ultimately, the future of exosome-based dermatology holds great promise, provided that collaboration, research, and innovation continue to be at the forefront. By embracing these exciting developments, the dermatological community can look forward to a future where exosomes play a central role in enhancing skin health, appearance, and overall well-being.

## Figures and Tables

**Table 1:** Common dermatological conditions, their characteristics, and underlying causes.

Condition	Description	Causes and Risk Factors
Acne Vulgaris [27]	Characterized by comedones, papules, pustules, nodules, and cysts, predominantly affecting the face, chest, and back.	Excess sebum production, follicular clogging, bacterial overgrowth (*C. acnes*), and inflammation.
Eczema (Dermatitis) [28]	A group of inflammatory skin disorders causing redness, itching, and irritation, including atopic, contact, and seborrheic dermatitis.	Genetic predisposition, immune system dysregulation, and environmental triggers (allergens, irritants).
Psoriasis [28]	A chronic autoimmune condition causing rapid skin cell buildup, leading to scales and erythematous plaques, often on the elbows, knees, and scalp.	Genetic susceptibility, immune system dysfunction, and environmental triggers (stress, infections).
Rosacea [29]	A chronic inflammatory skin disorder causing persistent facial redness, visible blood vessels, papules, and ocular symptoms.	Genetic factors, immune dysregulation, and environmental triggers (heat, alcohol, spicy food).
Skin Cancer [30]	Includes basal cell carcinoma, squamous cell carcinoma, and melanoma, presenting as abnormal moles, sores, or growths.	UV radiation exposure, genetic mutations, and immunosuppression.
Vitiligo [31]	Characterized by depigmented patches due to melanocyte loss.	Autoimmune attack on melanocytes and genetic predisposition.
Urticaria (Hives) [32]	Raised, itchy welts appearing suddenly, often as an allergic reaction.	Allergies (foods, medications), infections, stress, or idiopathic causes.
Fungal Infections [33]	Includes athlete’s foot, ringworm, and onychomycosis, causing skin peeling, itching, and nail discoloration.	Dermatophytes (*T. rubrum*, *M. canis*), yeast (*Candida* spp.), and warm and humid environments.
Hair Disorders [34]	Includes alopecia areata, hirsutism, and male-pattern baldness, leading to hair loss or excessive hair growth.	Autoimmune reactions, hormonal imbalances, and genetic factors.
Nail Disorders [35]	Involves nail discoloration, thickening, and deformities due to infections or systemic diseases.	Fungal infections, trauma, psoriasis, and systemic illnesses.
Contact Dermatitis [28]	Skin inflammation triggered by irritants or allergens, causing redness and itching.	Direct exposure to irritants (chemicals, detergents) or allergens (nickel, latex).
Pruritus (Itchy Skin) [28]	Persistent itching due to underlying skin conditions or systemic diseases.	Dermatitis, kidney disease, liver dysfunction, and neuropathic causes.

**Table 2:** Key exosome-based products and their clinical applications [45,46,47,48].

Product Name	Company	Source of Exosomes	Clinical Application	Key Benefits
Plated Intense Serum	Rion Aesthetics	Platelet-derived exosomes	Anti-aging, skin rejuvenation	Reduces facial redness, improves skin tone and texture
Renewosome™ Exosome Serums	(plated)™ Skin Science	Platelet-derived exosomes	Skin and hair rejuvenation	Promotes youthful appearance, supports skin and hair health
ELEVAI enfinity	ELEVAI Skincare	Not specified	Professional-grade skin treatment at home	Combines high-quality exosomes with potent ingredients for enhanced results
EXO-SKIN™ Exosome Serum	Dp Dermaceuticals	Wharton’s Jelly-derived MSC exosomes	Wound healing, skin rejuvenation	Accelerates wound closure, reduces scar formation
Cellese Exosome Products	Cellese	Not specified	Skin rejuvenation	Supports skin longevity, suitable for all skin types
Licorice Exosome Brightening Treatment Orbs	Snow Fox Skincare	Plant-derived exosomes (licorice root)	Hyperpigmentation, skin brightening	Lightens dark spots, evens out skin tone
CICA Exosome Replenishing Treatment Orbs	Snow Fox Skincare	Plant-derived exosomes (Centella Asiatica)	Post-treatment skin recovery, hydration	Soothes redness, enhances skin hydration

**Table 3:** Genetic transfection using exosomes vs. traditional methods.

Feature	Exosome-Mediated Transfection	Viral Transfection (e.g., Lentivirus, AAV)	Non-Viral Transfection (e.g., Liposomes, Electroporation)
Biocompatibility [45]	High; derived from host cells, reducing toxicity	Moderate; potential cytotoxicity due to viral components	Moderate to low; chemical agents may cause toxicity
Immunogenicity [49]	Low; minimal immune response	High; risk of immune activation and inflammation	Low to moderate; dependent on delivery system
Targeting Efficiency [50]	High; natural ability to target specific cells	Moderate; depends on viral vector engineering	Variable; depends on chemical modifications
Genetic Cargo Capacity [51]	Moderate; limited by exosome size (30–150 nm)	High; large DNA/RNA payload capacity	High; but may require complex formulations
Risk of Genomic Integration [52]	None; does not alter host genome	High; may integrate into the host genome, causing mutations	None; transient gene expression
Stability in Circulation [53]	High; stable in biological fluids and resistant to degradation	Moderate; may require modification for prolonged stability	Low to moderate; prone to enzymatic degradation
Production Scalability [54]	Challenging; requires optimized isolation techniques	High; well-established large-scale production	High; scalable for clinical applications
Regulatory Approval Complexity [55]	Lower; naturally derived, fewer safety concerns	High; stringent regulations due to genomic integration risk	Moderate; dependent on chemical formulation and safety profile

**Table 4:** Patent landscape of exosome-based products.

Patent Number	Title	Assignee/Inventor	Focus Area	Key Innovations
US20200331271A1	Compositions and Methods for Treating Skin Aging	Kimera Labs Inc.	Anti-aging and skin regeneration	Exosome-based formulations for collagen synthesis and wrinkle reduction
US10576136B2	Exosome-Based Drug Delivery System	Codiak Biosciences Inc.	Targeted drug delivery	Engineering exosomes for precise therapeutic payload delivery
US11324768B2	Methods for Enhancing Wound Healing	RoosterBio Inc.	Wound healing and tissue regeneration	Exosome compositions promoting faster tissue repair
EP3567892A1	Exosome-Based Cosmetic Composition	Medytox Inc.	Skincare and dermatology	Exosome-derived skincare formulations for hydration and elasticity improvement
WO2019229865A1	Method for Large-Scale Exosome Production	Exopharm Ltd.	Exosome isolation and purification	Scalable techniques for producing exosomes at high purity
CN110857767A	Exosome-Based Anti-Inflammatory Therapy	Ever Supreme Bio Technology	Dermatological inflammation	Exosome therapy for reducing inflammation in conditions like psoriasis
JP2020035462A	Nanovesicle Composition for Skin Rejuvenation	ExoCoBio Inc.	Anti-aging and pigmentation	Exosome-infused skincare formulations for anti-aging and skin brightening
US20210263159A1	Exosome Therapy for Hair Growth Stimulation	Direct Biologics LLC	Hair regeneration	Exosome compositions promoting follicular growth and hair restoration

**Table 5:** Future prospects and potential areas for further investigation in the field of exosomes in dermatology.

Future Directions	Description
Standardization of Exosome Isolation and Characterization	Establishing standardized protocols for the isolation and characterization of exosomes is crucial. This will ensure consistent quality and safety in exosome-based products, making them more accessible for clinical use.
Optimization of Exosome Cargo Loading	Research on enhancing the loading of exosomes with specific therapeutic cargo, such as nucleic acids or proteins, will allow for more targeted and effective treatments.
Personalized Treatment Approaches	Tailoring exosome-based therapies to individual patient profiles is a promising direction. Treatments can be optimized for each patient’s unique needs by considering genetic variations, skin types, and specific dermatological conditions.
Combination Therapies	Exploring the synergistic effects of exosome-based therapies with other dermatological treatments, such as laser therapy, microneedling, or traditional pharmaceuticals, can lead to more comprehensive and effective regimens for specific conditions.
Long-Term Safety Studies	Comprehensive, long-term safety studies are necessary to assess the impact of repeated exosome treatments on skin health, especially for chronic dermatological conditions and aesthetic procedures with extended regimens.
Regulatory Frameworks and Approvals	Collaborations between researchers, clinicians, and regulatory bodies are essential for establishing clear guidelines and approvals for exosome-based dermatological therapies, promoting patient safety and treatment efficacy.
Patient Education and Informed Consent	As exosome-based therapies gain traction, patient education and informed consent are paramount. Patients must understand the nature of these treatments, potential outcomes, and any associated risks.
Cost-Efficiency and Accessibility	Addressing the cost-effectiveness and accessibility of exosome-based therapies is crucial. Research into more economical production methods and insurance coverage for treatments can help make these therapies widely available.
Ethical Considerations	Ethical concerns surrounding the sourcing of exosomes, especially those derived from stem cells, require ongoing attention. Establishing ethical standards for exosome production is integral.
Collaboration and Multidisciplinary Research	Collaborative efforts between dermatologists, cell biologists, material scientists, and bioengineers can lead to breakthroughs in exosome-based therapies. A multidisciplinary approach fosters innovation and holistic understanding.
